# Trait mindfulness buffers depersonalization symptoms among young adults exposed to childhood abuse

**DOI:** 10.3389/fpsyg.2026.1838217

**Published:** 2026-07-09

**Authors:** Farah A. Mili, Emily K. Lindsay

**Affiliations:** Complementary Health Interventions Laboratory, Department of Psychology, University of Pittsburgh, Pittsburgh, PA, United States

**Keywords:** acceptance, childhood abuse, childhood trauma, depersonalization, mindfulness

## Abstract

**Introduction:**

Childhood abuse has a significant impact on adulthood mental health. Many children cope with the threat of abuse through avoidant strategies; however, this adaptation may contribute to the development of depersonalization symptoms and dissociative disorders. In contrast, the development of trait mindfulness, the tendency to monitor present-moment experience from an accepting perspective, may buffer depersonalization symptoms. This study tests whether trait mindfulness, particularly acceptance, moderates the association between childhood abuse and depersonalization among young adults with a self-reported history of abuse.

**Methods:**

As part of a larger trial, 81 participants (18–29 years, 85% female) reported childhood abuse (physical, emotional, sexual), trait mindfulness subscales, and depersonalization symptoms.

**Results:**

Regression models showed an association between childhood abuse and depersonalization symptoms (*β* = 0.30, *p* = 0.04); trait mindfulness moderated this relationship, such that the association between abuse and depersonalization was weakest among those with high mindfulness (*β* = −0.05, *p* = 0.73). Participants reporting high Observe and high Nonjudge subscale scores showed the strongest buffering effect (*β* = −0.12, *p* = 0.62), whereas the abuse-depersonalization association remained among those with high Observe with low Nonjudge scores (*β* = 0.50, *p* = 0.038). There was no association between abuse and depersonalization among those with high Observe and high Nonreact scores (*β* = 0.11, *p* = 0.67) or, unexpectedly, high Observe and low Nonreact scores (*β* = 0.36, *p* = 0.27). However, high Nonjudge and Nonreact scores alone (*β* = 0.21, *p* = 0.36; *β* = 0.04, *p* = 0.80) attenuated the abuse-depersonalization association.

**Discussion:**

Trait mindfulness, particularly acceptance, may buffer against the development of depersonalization symptoms in trauma-exposed adults. Understanding this relationship informs theories on traumatic resilience and mindfulness-based therapies.

## 1 Introduction

Childhood trauma has enduring effects on cognitive, emotional, and physiological development, contributing to higher risk for psychopathology across the lifespan (Glaser, 2000; Miller et al., 2011; Nusslock and Miller, 2016). One pathway through which childhood trauma contributes to psychopathology is depersonalization, an avoidant coping strategy characterized by emotional numbing, detachment from one’s body or surroundings, and fragmented self-experience (Terr, 2003; Sierra and Berrios, 2000; Shilony and Grossman, 1993; McKeen et al., 2021). However, not all trauma exposure leads to depersonalization or psychopathology, leading to an interest in resilience factors that may interrupt this association (Bonanno, 2004), such as trait mindfulness. This study tests whether trait mindfulness, and mechanisms of attention monitoring and acceptance in particular, moderate the relationship between childhood abuse and adulthood depersonalization symptoms. Better investigating these pathways to psychopathology and potential protective factors can help inform intervention approaches in at-risk populations.

Exposure to physical, emotional, or sexual abuse in childhood is thought to shape long-term coping behaviors (Felitti et al., 1998) and contribute to psychopathology by altering learned fear responses and reinforcing avoidance of threatening stimuli (Hosseini Ramaghani et al., 2019; McLaughlin et al., 2014). Children who experience repeated threat learn to expect and adapt to trauma, often finding ways to escape reality as a form of self-protection (Laricchiuta et al., 2023). In the short term, dissociative strategies such as depersonalization may allow children an escape from overwhelming emotions and sensations, but in the long-term, depersonalization is associated with dissociative disorders (Terr, 2003; Shilony and Grossman, 1993) and psychopathology more broadly (Yanartas et al., 2015). However, childhood trauma is not always associated with depersonalization symptoms (Michal et al., 2007; Vonderlin et al., 2018; Simeon et al., 2001), leading to an interest in individual characteristics that may protect against the development of psychopathology (Zhu et al., 2019).

Trait mindfulness—a tendency for nonjudgmental awareness of present-moment experience (Kabat-Zinn, 1994)—may be one such resilience factor (Brown and Ryan, 2003; Whitaker et al., 2014). Mindfulness is thought to be an innate human capacity tied to temperament, with some people showing a stronger tendency for open and sustained awareness of experiences (Brown et al., 2007). The development of trait mindfulness has also been associated with early childhood environmental factors such as caregiver attachments and peer social interactions (Pepping and Duvenage, 2016; Warren et al., 2021), suggesting that trait mindfulness represents a malleable disposition that can be shaped by experience (Roeser and Eccles, 2015; Tomlinson et al., 2018; Warren et al., 2023). Although trait mindfulness can be assessed as a cohesive unidimensional construct, trait mindfulness is commonly operationalized as encompassing two interrelated components: attention monitoring and acceptance (Bishop et al., 2004; Lindsay and Creswell, 2017). First, mindfulness involves using attention to *monitor* internal and external experience (e.g., thoughts, emotions, body sensations, sights, sounds, smells), and is commonly indexed by the Five Facet Mindfulness Questionnaire (FFMQ) “Observing” subscale, which captures attention to present-moment sensory and experiential phenomena (Baer et al., 2006). Second, momentary experience is approached with an orientation of *acceptance*, openness, and noninterference, and is commonly indexed by FFMQ “Nonjudgment” and “Nonreactivity” subscales, which capture the absence of evaluative judgment and emotional reactivity toward experience. According to the Monitor and Acceptance Theory (MAT), these two interacting processes have distinct and synergistic effects on specific affective outcomes (Lindsay and Creswell, 2017). Monitoring without acceptance is thought to heighten sensitivity to affective experience; for trauma-exposed adults, monitoring internal emotional experience can be intolerable, triggering depersonalization as a dissociative escape, consistent with depersonalization’s theorized function as an avoidant coping response to overwhelming affect (Shilony and Grossman, 1993; Sierra and Berrios, 2000). In contrast, monitoring with acceptance (or potentially acceptance alone) is thought to regulate emotion (Lindsay and Creswell, 2017; Lindsay et al., 2018) and reduce the need for avoidant coping. Therefore, trait mindfulness—and acceptance skills especially—may buffer against the development of depersonalization following childhood trauma (Thompson et al., 2011).

Conceptually, depersonalization, as a self-protective and defensively motivated state, stands in contrast with trait mindfulness (Medford, 2012). Whereas mindfulness involves awareness, presence, and connection between mind and body, depersonalization reflects emotional intolerance and numbing, detachment from experience, and fragmentation of memories and experiences (Zerubavel and Messman-Moore, 2015; Sierra et al., 2005). Correlational research supports this model, demonstrating inverse correlations between trait mindfulness and depersonalization symptoms (Michal et al., 2007). However, mindfulness facets may differentially relate to depersonalization (Hunter et al., 2003). For example, monitoring alone has been correlated with dissociative tendencies (Levin et al., 2022), especially in the context of trauma exposure (Boughner et al., 2016). In contrast, awareness and openness to momentary experience together may reduce the tendency to default to maladaptive coping mechanisms such as depersonalization, and indeed, the combination of high trait monitoring and acceptance skills have been shown to associate with lower depersonalization symptoms compared to high trait monitoring with low acceptance (Levin et al., 2022). It is worth noting that mindful nonreactivity and depersonalization may superficially resemble each other in involving distance from experience. However, unlike depersonalization, an involuntary detachment and disconnection from experience (Sierra and Berrios, 2000), nonreactivity reflects a deliberate, accepting stance that allows experience to arise and pass without suppression or escape. Overall, the balance between monitoring and acceptance skills may be particularly important for depersonalization outcomes, yet childhood trauma may disrupt the developmental processes through which these skills are cultivated.

Childhood trauma may disrupt the development of—and predict individual differences in—mindfulness skills. Theoretical work focused on the link between childhood trauma and trait mindfulness paints a clear picture: children facing the existential psychological threat of abuse often use experiential avoidance as an escape, possibly because trauma leads children to distrust or disengage from emotional body sensations (in contrast to the interoceptive awareness mindfulness cultivates) before these experiences can be integrated into memory (Oldroyd et al., 2019; Bolduc et al., 2018). It may not be adaptive for children who grow up in threatening environments to develop and utilize mindfulness skills such as present-moment focus (Pepping and Duvenage, 2016) in the context of abuse; indeed, heightened interoceptive awareness may amplify the emotional intensity of traumatic experiences (Gibson, 2024). Chronic trauma exposure may therefore disrupt the development of brain regions foundational for interoceptive awareness and emotion processing (Gibson, 2024), undermining the cultivation of mindful awareness more broadly (Oldroyd et al., 2019). Correlational findings are consistent with this model, showing an association between adverse experiences such as childhood sexual abuse or interpersonal trauma and lower trait mindfulness overall (Daigneault et al., 2016; McKeen et al., 2021), as well as profiles marked by high monitoring and low acceptance (Hémond-Dussault et al., 2022). Overall, most studies support the idea that childhood trauma disrupts the development of trait mindfulness and the processes underlying its naturalistic development, but that when trait mindfulness does develop, it may moderate the association between childhood trauma and adulthood mental health symptoms (Vancappel et al., 2021; Vancappel et al., 2022).

In a sample of emerging adults exposed to moderate-to-severe abuse in childhood, this study tests whether trait mindfulness buffers against depersonalization symptoms. Based on prior theory and research, we hypothesize:

Hypothesis 1: More severe childhood abuse will associate with higher depersonalization symptoms.

Hypothesis 2: Higher trait mindfulness will attenuate the association between childhood abuse and depersonalization symptoms.

In exploratory analyses guided by MAT, we predict that high monitoring combined with high acceptance (high Observe with high Nonjudge or Nonreact) will buffer the association between childhood abuse and depersonalization symptoms, whereas high monitoring with low acceptance (high Observe with low Nonjudge or Nonreact) will not. Alternatively, high acceptance independent of monitoring skills may buffer this association. Results from the study will enhance understanding of the link between trait mindfulness and depersonalization in a sample exposed to childhood trauma, with implications for using mindfulness training to treat depersonalization symptoms stemming from early life trauma and abuse.

## 2 Methods

### 2.1 Participants

Participants were 81 young adults who enrolled in the ReMind Study conducted in Pittsburgh, with the present analyses focused on cross-sectional baseline data. The larger ReMind Study was a two-arm randomized controlled trial that aimed to test feasibility and acceptability of two-week remote mindfulness and coping interventions among emerging adults who recalled a history of childhood abuse (Lindsay et al., 2025a, 2025b). Data was collected from September 2022 until May 2024 when the recruitment goal was reached. Sample size for the larger trial was based on the pilot trial recommendation of 25–35 participants per arm.

Participants were recruited from the Pitt+Me research registry, which advertises to over 300,000 residents in the Pittsburgh area. To be eligible for the larger ReMind intervention trial, participants had to be fluent in English, own a smartphone, and report moderate-to-severe childhood physical (>9), emotional (>12), or sexual (>7) abuse on the Childhood Trauma Questionnaire (CTQ; Bernstein et al., 2003). Exclusion criteria for the larger ReMind trial included: diagnosis of a chronic physical, psychiatric, or neurological disorder; suicidal thoughts or wishes; systematic mind–body practice more than twice a week; medication use that affected HPA-axis, autonomic, inflammatory, or blood clot activity; current antibiotic, antiviral, or antimicrobial treatment; substance abuse; night shift work; or pregnancy.

The study was pre-registered with ClinicalTrials.gov and approved by the University of Pittsburgh Institutional Review Board (IRB), with the present cross-sectional analyses approved by the IRB with reference number MOD21030189-004. All participants provided written informed consent to participate in the full trial, and participant data was anonymized through ID numbers. Funding for the ReMind Study came from the National Institute of Health (NIH) and the Mind & Life Institute.

### 2.2 Procedure

The present analyses involve cross-sectional data collected in person at baseline. The larger ReMind trial also included a two-week intervention surrounded by baseline, post-intervention, and one-month follow-up assessments in the laboratory and in daily life. *N* = 1,097 participants were recruited and completed an online pre-screening. The 249 participants who met initial inclusion criteria, including thresholds for moderate-to-severe abuse on the CTQ, were invited to an in-person baseline visit; of the 137 participants who attended this visit, a final sample of *n* = 81 participants were determined eligible and enrolled (Lindsay et al., 2025a,b). Participants completed two questionnaire batteries, which included demographic characteristics, childhood trauma history (CTQ), trait mindfulness (FFMQ), and depersonalization symptoms (CDS) used in the present analyses. Due to an error, seven enrolled participants only met the CTQ inclusion thresholds at pre-screening, but not in person. Analyses run without these unique cases did not substantially change the pattern of results.

### 2.3 Materials

#### 2.3.1 Childhood abuse

The 28-item Childhood Trauma Questionnaire-Short Form (CTQ) was used to assess physical (ex. “I was punished with a belt, a board, a cord, or some other hard object”), emotional (ex. “I thought that my parents wished I had never been born”), and sexual abuse (ex. “Someone tried to make me do sexual things or watch sexual things”) during childhood (Bernstein et al., 2003). The three abuse subscales were comprised of five items each. The CTQ also includes three items assessing minimization/denial and two five-item subscales assessing physical and emotional neglect; these subscales were not included in analyses. Responses were collected using a Likert scale from 1 *(never true)* to 5 *(very often true)*, and relevant items were reverse-scored so that higher scores represent higher abuse exposure (Likert, 1932). The CTQ abuse score (15 items, *α* = 0.74), used in the present analyses, is a sum of the three abuse subscales (physical, emotional, sexual), giving equal weight to all forms of abuse (Bernstein et al., 2003); scores range from 15 to 75, with a score over 28 indicating severe exposure to abuse.

#### 2.3.2 Depersonalization symptoms

Sierra and Berrios’s (2000) 29-item Cambridge Depersonalization Scale (CDS) recorded the frequency and duration of depersonalization symptoms, which encompass experiences of feeling detached from the self and includes side effects such as emotional numbing and experiential distortion, within the past month (ex. “What I see looks ‘flat’ or ‘lifeless’, as if I were looking at a picture,” “I have the feeling of being outside my body”). Frequency of each item was assessed with a 0 *(never)* to 4 *(all the time)* Likert scale. For questions with a frequency score greater than 0, participants also rated the duration of the symptoms from 1 *(few seconds)* to 6 *(more than a week)*. Both scores were summed to calculate total CDS score (29 items, *α* = 0.96). Higher scores indicated more severe depersonalization symptoms, with a score of 70 and above associating with clinical depersonalization disorder.

#### 2.3.3 Trait mindfulness

The Five Facet Mindfulness Questionnaire-Short Form (FFMQ; Bohlmeijer et al., 2011; Baer et al., 2006) is a 24-item scale assessing trait mindfulness, or the ability to step back from and neutrally observe present-moment thoughts, feelings, and emotions. The five mindfulness facets include Observe (ex. “I pay attention to physical experiences, such as the wind in my hair or sun on my face”), Nonjudge (ex. “I tell myself I should not be feeling the way I’m feeling,” reverse scored), Nonreact (ex. “I watch my feelings without getting carried away by them”), Describe (ex. “I can easily put my beliefs, opinions, and expectations into words”), and ActAware (ex. “I find myself doing things without paying attention,” reverse scored). The Observe subscale includes 4 items, and all other subscales include five items. Items are scored on a Likert scale from 1 *(never or very rarely true)* to 5 *(very often or always true)*, with relevant items reverse-scored so that higher scores indicate higher trait mindfulness. Subscale responses were averaged. All items were averaged to calculate the overall trait mindfulness score used in the present analyses (24 items, *α* = 0.87). Additionally, sensitivity analyses testing Monitor and Acceptance Theory predictions focused on Observe (4 items, α = 0.73) and Nonreact (5 items, α = 0.72) and Nonjudge (5 items, α = 0.82) subscales. Consistent with MAT, the Observe subscale indexes attentional monitoring and the Nonjudge and Nonreact subscales index acceptance-related orientations toward experience (Lindsay and Creswell, 2017).

#### 2.3.4 Analyses

Analyses included all available data and were conducted using Jamovi (R core team, 2021; Version 2.5; The jamovi project, 2024). Prior to analysis, scores for CTQ abuse, CDS, and FFMQ were examined for outliers, and internal reliability for each measure was assessed with Cronbach’s alpha. Preliminary analyses characterized the sample and examined correlations between sample characteristics and primary variables to evaluate covariates (age, race, ethnicity, sex; history of mental health diagnoses of depression, anxiety, or panic disorder; BMI; student status; Kim, 2015).

Primary analyses were run (1) without adjusting for covariates and (2) adjusting for covariates related to the primary variables. The first hypothesis, that childhood abuse will be positively correlated with depersonalization symptoms, was tested using simple linear regression (Fox and Weisberg, 2020; Gallucci, 2019). To investigate the second hypothesis, that trait mindfulness will moderate the effect of childhood abuse on depersonalization symptoms, multiple regression analyses tested childhood abuse, trait mindfulness, and their interaction (childhood abuse × trait mindfulness) as predictors of depersonalization symptoms, with CTQ abuse and FFMQ scores centered on the grand mean (Aiken et al., 1991). Follow-up simple slope analyses compared the connection between CTQ and CDS scores at one standard deviation above and below the mindfulness mean. Regression assumptions were assessed prior to analysis. A Shapiro–Wilk test indicated deviation from normality of residuals (*p* = 0.003), and visual inspection of Q-Q plots suggested mild skew. Because regression estimates are generally robust to non-normality but standard errors may be affected, analyses were conducted using robust standard errors. Results were substantively unchanged. Multicollinearity was low (variable inflation factor values ranged from 1.04 to 1.17).

To conduct exploratory analyses testing MAT predictions, participants were grouped into categories based on their FFMQ subscale scores—(1) high (at or above the mean) Observe with high Nonjudge (*n* = 21) or Nonreact scores (*n* = 22), (2) high Observe with low (below the mean) Nonjudge (*n* = 16) or Nonreact scores (*n* = 25), (3) low Observe with high Nonjudge (*n* = 15) or Nonreact scores (*n* = 19), and (4) low Observe with low Nonjudge (*n* = 29) or Nonreact (*n* = 25) scores. Specifically, general linear model analyses focused on the simple slopes of high Observe (high monitoring) and high Nonjudge or Nonreact (high acceptance) vs. high Observe (high monitoring) and low Nonjudge or Nonreact (low acceptance) groups to test whether high acceptance buffers the association between childhood abuse and depersonalization symptoms at high monitoring. Analyses testing the alternative MAT prediction, that acceptance alone might moderate this relation, grouped participants into high (above the mean) or low (at or below the mean) Nonjudge (high: *n* = 36, low: *n* = 45) or Nonreact (high: *n* = 41, low: *n* = 40), independent of Observe. General linear models tested associations between CTQ abuse score and CDS score at high vs. low Nonjudge or Nonreact.

## 3 Results

### 3.1 Preliminary analyses

Participants were emerging adults, 85.2% female; 48.1% white, 28.4% Asian, 17.3% black; and 6.2% Hispanic/Latino (see Table 1 for baseline characteristics). 70% of participants reported moderate-to-severe emotional abuse, 27% reported moderate-to-severe physical abuse, and 27% reported moderate-to-severe sexual abuse. There were no significant correlations between potential covariates and primary variables (Supplementary Table 1). As such, adjusted analyses included only basic demographic characteristics (age, sex, race, and ethnicity).

**Table 1:** Sample characteristics.

Variable	M (SD) or *n* (%)
Age in years	23.8 (3.28)
Sex (female)	69 (85.2%)
Gender	
Female	61 (75.3%)
Male	12 (14.8%)
Non-binary/Genderqueer	8 (9.9%)
Race	
White/Caucasian	39 (48.1%)
Asian	23 (28.4%)
Black/African American	14 (17.3%)
Bi- or multi-racial	4 (4.9%)
Other	1 (1.2%)
Ethnicity (not Hispanic or Latino)	76 (93.8%)
BMI	27.9 (7.90)
Depression, anxiety, or panic disorder history (yes)	42 (51.9%)
Student status (yes)	48 (59.3%)
Childhood Trauma Questionnaire abuse score	29.0 (6.95)
Emotional abuse score	14.19 (0.44)
Physical abuse score	7.90 (0.41)
Sexual abuse score	6.99 (0.43)
Five Facet Mindfulness Questionnaire score	3.1 (0.52)
Observe facet score	3.6 (0.84)
Nonjudge facet score	2.8 (0.67)
Nonreact facet score	3.0 (0.90)
Cambridge Depersonalization Scale score	55.3 (45.1)

### 3.2 Primary analyses

#### 3.2.1 Hypothesis 1

A linear regression model supported the hypothesis that childhood abuse would associate with depersonalization symptoms (*β* = 0.27, *SE* = 0.98, *p* = 0.079; η^2^_p_ = 0.07), such that more severe abuse in childhood associated with higher depersonalization symptoms. This association held when adjusting for demographic covariates (age, sex, race, ethnicity) (*β* = 0.30, *SE* = 0.94, *p* = 0.044; η^2^_p_ = 0.09; Supplementary Figure 1).

#### 3.2.2 Hypothesis 2

Multiple regression analyses supported the hypothesis that trait mindfulness would moderate the association between childhood trauma and depersonalization symptoms; there was a significant association between the interaction term, CTQ abuse score × FFMQ score, and depersonalization in unadjusted (*β* = −0.25, *SE* = 1.14, *p* = 0.007, η^2^_p_ = 0.08) and covariate-adjusted models (*β* = −0.25, *SE* = 1.19, *p* = 0.012, η^2^_p_ = 0.08; Supplementary Table 2).

**Figure 1:**
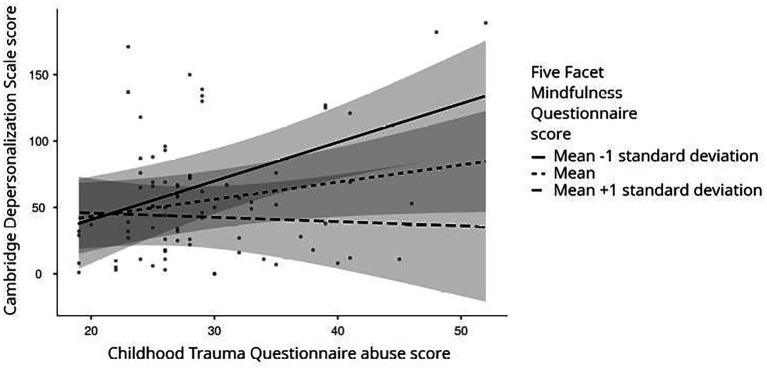
Moderating effect of mindfulness on the relation between childhood abuse and depersonalization scores.

Simple slopes analysis showed no association between CTQ and CDS when FFMQ score is at its mean (unadjusted: *β* = 0.17, *SE* = 0.67, *p* = 0.109; covariate-adjusted: *β* = 0.20, *SE* = 0.67, *p* = 0.056) or one standard deviation above the mean (unadjusted: *β* = −0.09, *SE* = 0.91, *p* = 0.535; covariate-adjusted: *β* = −0.05, *SE* = 0.92, *p* = 0.727), but a significant association at one standard deviation below the FFMQ mean (unadjusted: *β* = 0.42, *SE* = 0.88, *p* = 0.003; adjusted: *β* = 0.45, *SE* = 0.91, *p* = 0.002), supporting a buffering effect of moderate-to-high trait mindfulness on the link between childhood abuse and depersonalization symptoms (Supplementary Table 2; Figure 1).

### 3.3 Exploratory hypotheses

#### 3.3.1 Observe and nonjudge

**Figure 2:**
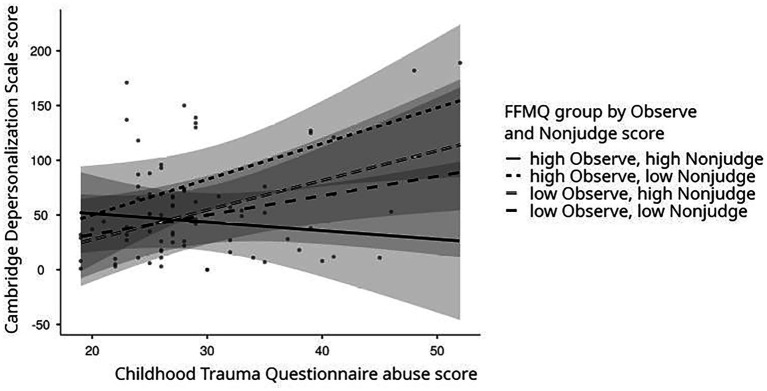
Moderating effect of Observe and Nonjudge mindfulness facets on the relation between childhood abuse and depersonalization scores. FFMQ, Five Facet Mindfulness Questionnaire.

Based on MAT, a general linear model should present a significant association between CTQ and CDS at high Observe (monitoring) and low Nonjudge (acceptance) scores, and no association when both Observe and Nonjudge scores are high. Supporting the hypothesis, there was a significant association at high Observe and low Nonjudge (*β* = 0.504, *p* = 0.038), but not at high Observe and high Nonjudge (*β* = −0.120, *p* = 0.623), such that Nonjudge buffered the association between Observe score and depersonalization symptoms (Figure 2; Table 2).

**Table 2:** Simple slopes for the moderating effects of observe, nonjudge, and their interaction on the childhood abuse-depersonalization association.

Mindfulness facets	*B*	Standard error	β	*p*
Observe and Nonjudge				
High Observe, high Nonjudge	−0.78	1.58	−0.120	0.623
High Observe, low Nonjudge	3.27	1.55	0.504	0.038*
*Low Observe, high Nonjudge*	*2.71*	*1.78*	*0.417*	*0.132*
*Low Observe, low Nonjudge*	*1.78*	*1.43*	*0.273*	*0.218*
Nonjudge only				
High Nonjudge	1.34	1.45	0.207	0.356
Low Nonjudge	2.82	1.10	0.434	0.012*

Italicized rows represent slopes that were not included in hypotheses. *p < 0.05, **p < 0.01.

The alternative MAT theory predicted that childhood abuse and depersonalization symptoms should be associated at low levels of Nonjudge, but not at high levels, independent of Observe. The general linear model supported this hypothesis (low Nonjudge: *β* = 0.434, *p* = 0.012; high Nonjudge: *β* = 0.207, *p* = 0.356) and demonstrated a buffering effect of Nonjudge across all Observe scores (Supplementary Figure 2; Table 2), although Nonjudge was a stronger buffer when coupled with high Observe skills, supporting the original MAT hypothesis.

#### 3.3.2 Observe and nonreact

A general linear model was run with Observe and Nonreact groups, also predicting a significant slope (i.e., the relation between CTQ and CDS) among participants with high Observe and low Nonreact scores, but not high Observe and high Nonreact scores. Although, as predicted, there was no significant slope at high Observe and high Nonreact scores (*β* = 0.113, *p* = 0.672), contrary to prediction, there was also not a significant slope at high Observe and low Nonreact scores (*β* = 0.359, *p* = 0.272). Thus, Nonreact was not shown to interact with Observe to buffer the association between childhood abuse and depersonalization symptoms (Supplementary Figure 3, Table 3).

**Table 3:** Simple slopes for the moderating effects of Observe, Nonreact, and their interaction on the childhood abuse-depersonalization association.

Mindfulness facets	*B*	Standard error	*β*	*p*
Observe and Nonreact				
High Observe, high Nonreact	0.73	1.72	0.113	0.672
High Observe, low Nonreact	2.33	2.11	0.359	0.272
*Low Observe, high Nonreact*	*−0.99*	*0.71*	*−0.152*	*0.167*
*Low Observe, low Nonreact*	*4.16*	*1.34*	*0.641*	0.003**
Nonreact only				
High Nonreact	0.28	1.12	0.043	0.804
Low Nonreact	3.48	1.14	0.536	0.003**

Italicized rows represent slopes that were not included in hypotheses. *p < 0.05, **p < 0.01.

The alternative MAT hypothesis was supported, with a significant association between childhood trauma and depersonalization symptoms at low Nonreact (*β* = 0.536, *p* = 0.003) but not high Nonreact (*β* = 0.043, *p* = 0.804). Indeed, the buffering effect of Nonreact was stronger independent of Observe (high Nonreact: *β* = 0.043; high Observe, high Nonreact: *β* = 0.113; Supplementary Figure 4; Table 3), providing more support for the alternative MAT prediction.

## 4 Discussion

The present study examined whether trait mindfulness attenuates the association between childhood abuse exposure and depersonalization symptoms in young adulthood. Consistent with previous findings, abuse severity associated with depersonalization symptoms in this trauma-exposed sample. Trait mindfulness moderated this relationship, such that childhood abuse severity did not associate with depersonalization symptoms among those with higher trait mindfulness. Further, the mechanistic interaction between trait monitoring and acceptance skills explained this buffering effect, with acceptance demonstrating a more powerful buffer against depersonalization symptom development than trait mindfulness as a broad construct. Findings suggest that developing mindfulness skills, especially an orientation of acceptance toward experience, may protect against the development of depersonalization symptoms. Trait mindfulness—and acceptance in particular—may reduce the tendency to use experiential avoidance strategies and instead encourage reality anchoring and emotion regulation.

Results were largely consistent with Monitor and Acceptance Theory (MAT), which posits that trait attention monitoring and acceptance skills play distinct and synergistic roles in psychological well-being (Lindsay and Creswell, 2017). Exploratory findings provided some evidence that the association between abuse exposure and depersonalization was strong among those reporting high trait monitoring without concomitant acceptance, whereas both monitoring and acceptance traits together buffered the link between childhood trauma and depersonalization symptoms. Trait acceptance also buffered this link independent of monitoring, although the link between abuse and depersonalization was weaker among those with both high monitoring (Observe) and high acceptance (Nonjudge) skills. Acceptance may allow people to notice rather than avoid distressing sensations or emotions, thereby reducing the likelihood of emotional numbing or detachment. These results support the idea that monitoring by itself—without acceptance—intensifies experience in ways that lead to a tendency to dissociate from that experience, whereas monitoring with acceptance fosters engagement with, rather than withdrawal from, internal experience.

These findings aligned with both MAT predictions *and* the alternative MAT prediction—namely, that trait acceptance skills predict psychological health (in this case, depersonalization symptom severity) independent of monitoring skill. Supporting original MAT predictions, Nonjudge and Observe subscales interacted to buffer the association between childhood trauma and depersonalization symptoms. Further, findings did not show this same buffering effect among people with high Nonjudge and low Observe traits, suggesting important contributions of monitoring. However, supporting alternative predictions, Nonreact alone buffered the trauma-depersonalization association, joining other studies showing associations between trait acceptance alone and lower mental health symptoms (Lindsay and Creswell, 2017). Prior research on Nonreact scores has yielded inconsistent associations with depersonalization and related symptoms (Levin et al., 2022; Baer et al., 2006), perhaps due to overlap between Nonreact and depersonalization constructs in non-meditator samples. Indeed, items such as “I can just notice [distressing thoughts or images] without reacting” may read differently depending on the sample; in trauma-exposed individuals without meditation experience, endorsement of such items may reflect dissociative detachment rather than the deliberate, accepting stance that characterizes mindful nonreactivity, potentially attenuating its buffering effect and contributing to inconsistent findings across samples. Importantly, this study may not have been adequately powered to examine interactions between monitoring and acceptance skills; categorizing people into ‘high’ vs. ‘low’ monitoring and acceptance yielded small subgroups, with reduced ability to detect consistent patterns on these continuously measured variables. Nonetheless, in this sample, acceptance skills, and possibly their interaction with monitoring skills, appear to be important components of trait mindfulness in buffering the development of depersonalization symptoms.

Findings should be interpreted with consideration of sample characteristics; the current sample was intentionally recruited for moderate-to-severe abuse exposure, a robust risk factor for depersonalization symptoms and psychopathology more broadly (Terr, 2003; Shilony and Grossman, 1993; Yanartas et al., 2015). As expected, study participants reported higher rates of childhood abuse (Bernstein et al., 2003) and depersonalization symptoms (Sierra and Berrios, 2000) and lower levels of trait mindfulness (Baer et al., 2008) relative to community norms. Exposure to chronic threat in childhood may disrupt the development of acceptance-related skills (Pepping and Duvenage, 2016), instead leaving people more vulnerable to experiential avoidance and detachment. Given this developmental context, findings suggest that the capacity for mindfulness may be a particularly important protective factor among trauma-exposed adults. Ultimately, by attenuating the effects of abuse on depersonalization symptoms, the development of mindfulness in this population may promote long-term resilience to psychopathology. Because experiential avoidance and disrupted acceptance skills are not unique to trauma-exposed populations, these MAT findings may have broader relevance for clinical populations with similar tendencies toward experiential avoidance.

Although this study focused on trait mindfulness, it may also have implications for mindfulness-based treatment of depersonalization symptoms that can arise following childhood trauma. Indeed, 8-week mindfulness interventions have shown promise for reducing symptoms among adults who report a history of childhood trauma (for a review, see Joss and Teicher, 2021). Identifying which facets of trait mindfulness buffer psychopathological symptoms may inform which components of mindfulness training to emphasize for those with trauma histories and low trait mindfulness. Traditionally, mindfulness interventions first introduce monitoring practices to train attention toward present-moment experience, which can increase contact with emotional and sensory experiences and counteract avoidant, dissociative tendencies (Lindsay and Creswell, 2017). At the same time, this increased contact with experience can intensify negative affect following brief meditation in trauma-exposed samples (Zhu et al., 2019). Given that high monitoring skills are associated with negative affect in meditation-naïve samples, baseline mindfulness traits might interact with meditation training, with high monitoring and low acceptance traits escalating negative affect in early stages before acceptance skills are developed (Blacker et al., 2011). In these early stages of training, meditation practices that emphasize acceptance or lovingkindness may reduce sensory or emotional overwhelm in trauma samples, who are at risk for traumatic re-experiencing and other distressing experiences that may trigger depersonalization (Britton et al., 2021; Joss and Teicher, 2021). Mindfulness interventions adapted for trauma-exposed populations that focus on cultivating a felt sense of safety and normalizing reactions to trauma (Kelly, 2015) have shown promise for reducing trauma symptoms (Aizik-Reebs et al., 2021; Goldsmith et al., 2014; Kim et al., 2013; Treleaven, 2018). Trait mindfulness thus functions as a protective factor against depersonalization symptoms, yet the process of developing mindfulness skills through intervention may initially carry risk for trauma-exposed individuals before acceptance skills are established, warranting caution in early stages of training. With trauma-sensitive adaptation, however, mindfulness interventions represent a promising long-term approach for reducing depersonalization symptoms in this population.

At the same time, how the trait mindfulness results observed here translate to mindfulness intervention recommendations is still speculative. Ultimately, even though mindfulness interventions have been shown to boost trait mindfulness in adult (Kiken et al., 2015) and child/adolescent samples (Porter et al., 2022), the naturalistic development of trait mindfulness in childhood may be cultivated through mechanisms tied to temperament, interoceptive development, and secure caregiving relationships (Gibson, 2024; Farb et al., 2015), distinct from the monitoring and acceptance skills trained through formal meditation practice. Whether the development of trait mindfulness through mindfulness intervention would translate into reduced depersonalization symptoms among adults exposed to childhood trauma remains unclear; if developing monitoring and acceptance skills naturalistically vs. through intervention have equivalent effects on depersonalization, at least 8 weeks of mindfulness training may produce the trait mindfulness gains (Baer et al., 2012) needed to achieve comparable reductions in depersonalization symptoms. Further mindfulness intervention research is needed to understand how monitoring and acceptance skills develop and interact to impact depersonalization, dissociation, and mental health symptoms in childhood trauma samples, appropriate intervention dosing, and how mindfulness intervention content can be tailored to baseline trait mindfulness levels.

The study has several notable limitations. First, the sample of 81 was relatively small, so findings should be interpreted with caution. Second, childhood trauma is a risk factor for severe psychiatric conditions, substance abuse, and suicidality, all of which were exclusion criteria for the study; excluding these vulnerable populations may limit insight into those at greatest risk (Alkema et al., 2024). Third, given the uneven distribution of female over male participants, findings may be limited in their generalizability to the larger population of adults who recall childhood abuse exposure, where sex differences are often reported for sexual abuse (Stoltenborgh et al., 2014) and sex and trauma have been shown to interact to affect brain development (Badura-Brack et al., 2020). Fourth, the retrospective self-report Childhood Trauma Questionnaire does not capture important details about the frequency or severity of abuse (McDonald and Calhoun, 2010) or when, developmentally, it took place. Future studies that use interview-based measures of childhood trauma (for example, the Childhood Experiences of Care and Abuse interview; Bifulco et al., 1994) are needed to clarify how these characteristics of trauma impact the development of both trait mindfulness and depersonalization symptoms. Since trait mindfulness has been linked to attachment processes and caregiver bonds in early childhood (Pepping and Duvenage, 2016), one possibility is that preverbal trauma or abuse by a trusted adult (as opposed to a stranger) may be most disruptive to the development of trait mindfulness. Future research is needed to examine the impact of trauma experienced at different developmental periods, the chronicity of trauma exposure, and the potential interaction effect of sex and differences in brain maturation. Finally, considering childhood abuse as a unipolar facet may also lose some sensitivity (Hodgdon et al., 2018), as emotional abuse and neglect have been shown to have stronger correlations with lower mindfulness levels (de Moraes et al., 2023; Michal et al., 2007) and depersonalization symptom severity (Michal et al., 2007; Simeon et al., 2001) than overall childhood trauma or abuse. Conversely, the focus on depersonalization rather than dissociative symptoms more broadly may limit generalization. However, consistent with the present findings, acceptance and attention have been previously identified as mediators in the negative relationship between trait mindfulness and dissociative experiences more broadly (Vancappel et al., 2021). Further research is warranted to develop and test mindfulness interventions for addressing depersonalization symptoms in abuse populations.

## 5 Conclusion

Overall, findings suggest that trait mindfulness, and acceptance in particular, is a resilience factor that might reduce the development of depersonalization symptoms and ultimately dissociative psychopathology among people with a history of childhood trauma. As hypothesized, childhood abuse was related to depersonalization symptoms, and trait mindfulness, especially monitoring with acceptance, attenuated this association. The present study contributes to a mechanistic understanding of how trait mindfulness may help reduce depersonalization symptoms and build resilience following childhood abuse, offering insight for mindfulness intervention efforts.

## Data Availability

The data analyzed in this study is subject to the following licenses/restrictions: data was provided from an ongoing study through an IRB amendment. Requests to access these datasets should be directed to Emily K. Lindsay, emily.lindsay@pitt.edu.
