# Supplementary figures and images for: Trait mindfulness buffers depersonalization symptoms among young adults exposed to childhood abuse
Source: Front Psychol. 2026 Jul 9;17:1838217. doi: 10.3389/fpsyg.2026.1838217 (PMC13391278; doi:10.3389/fpsyg.2026.1838217)

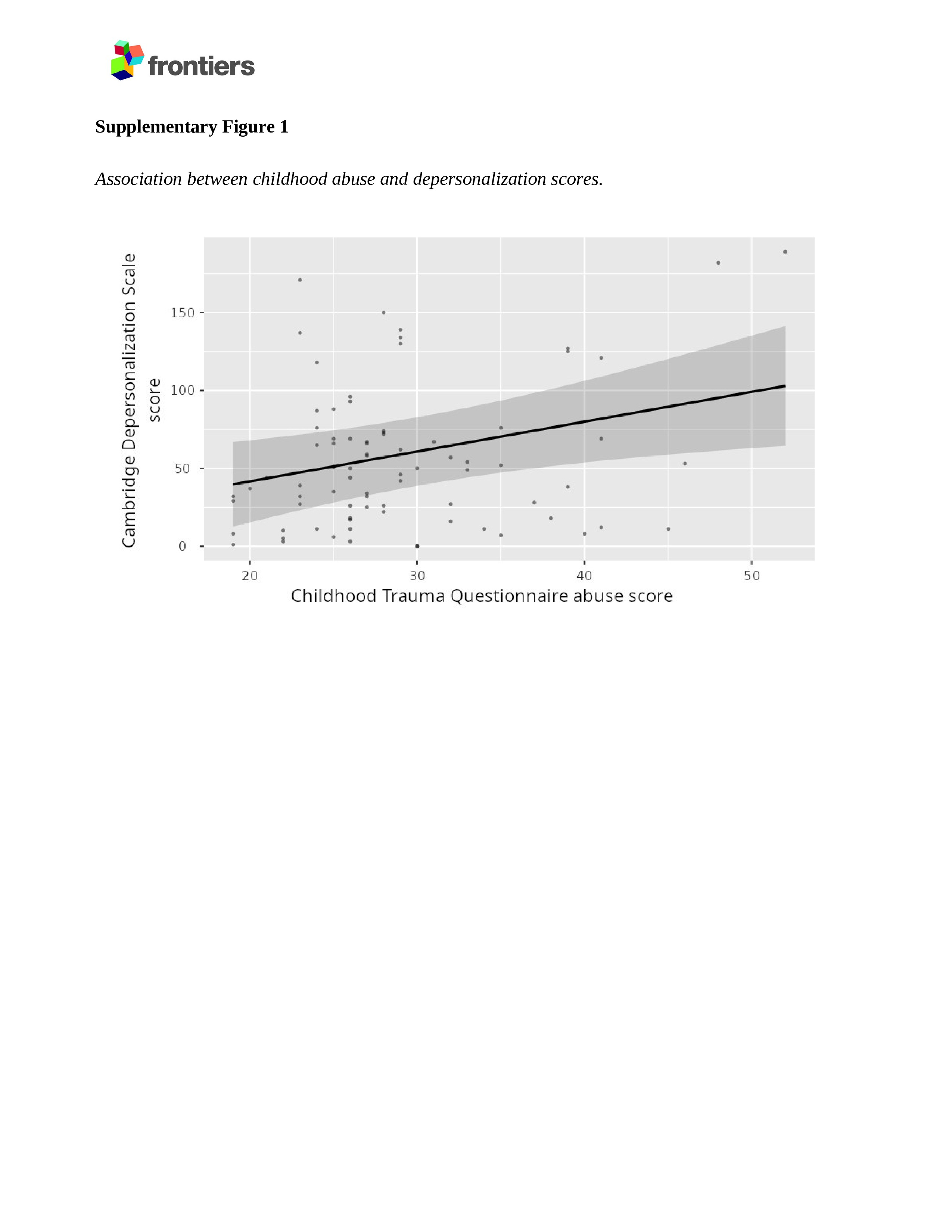

Supplement: Supplementary file 1 [file Image_1.JPEG]

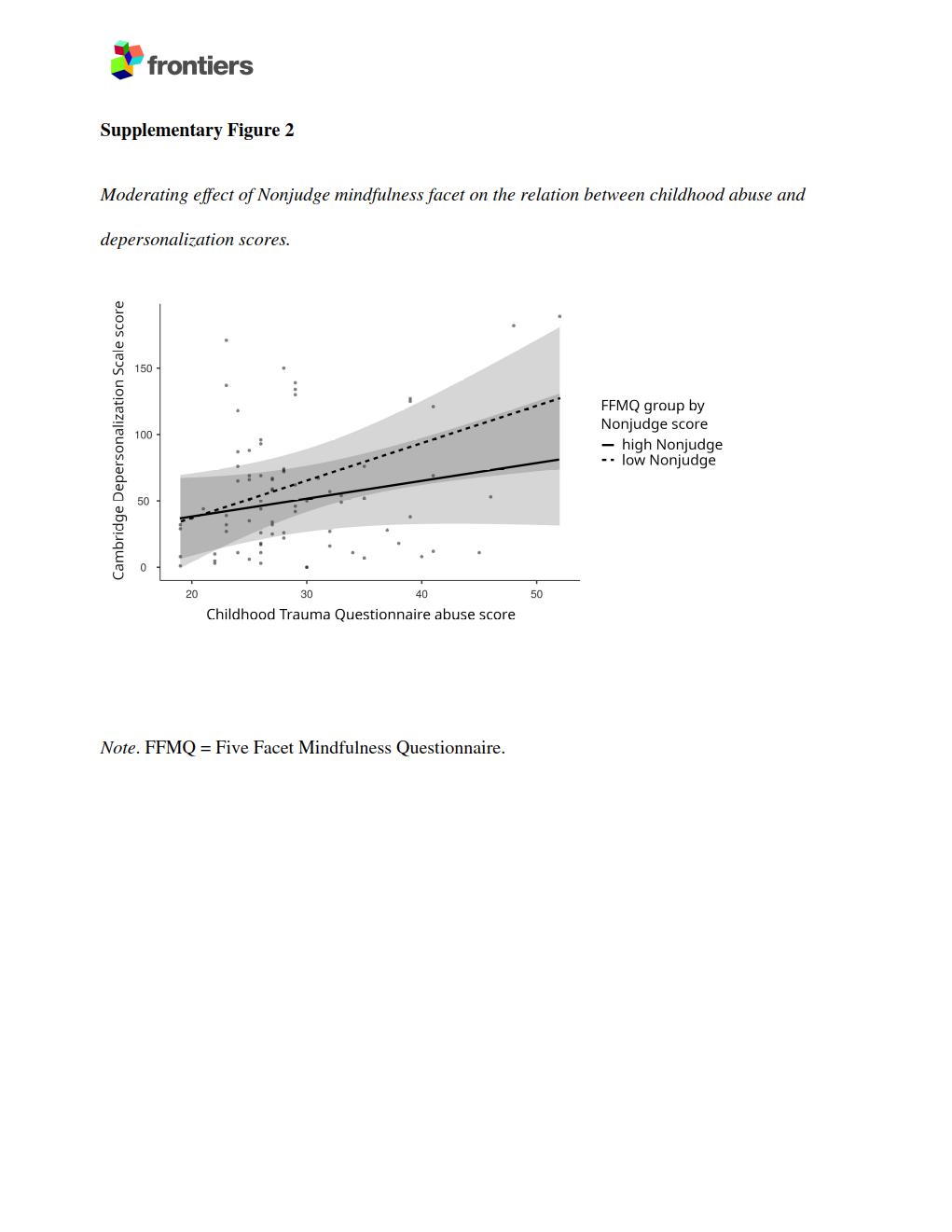

Supplement: Supplementary file 2 [file Image_2.JPEG]

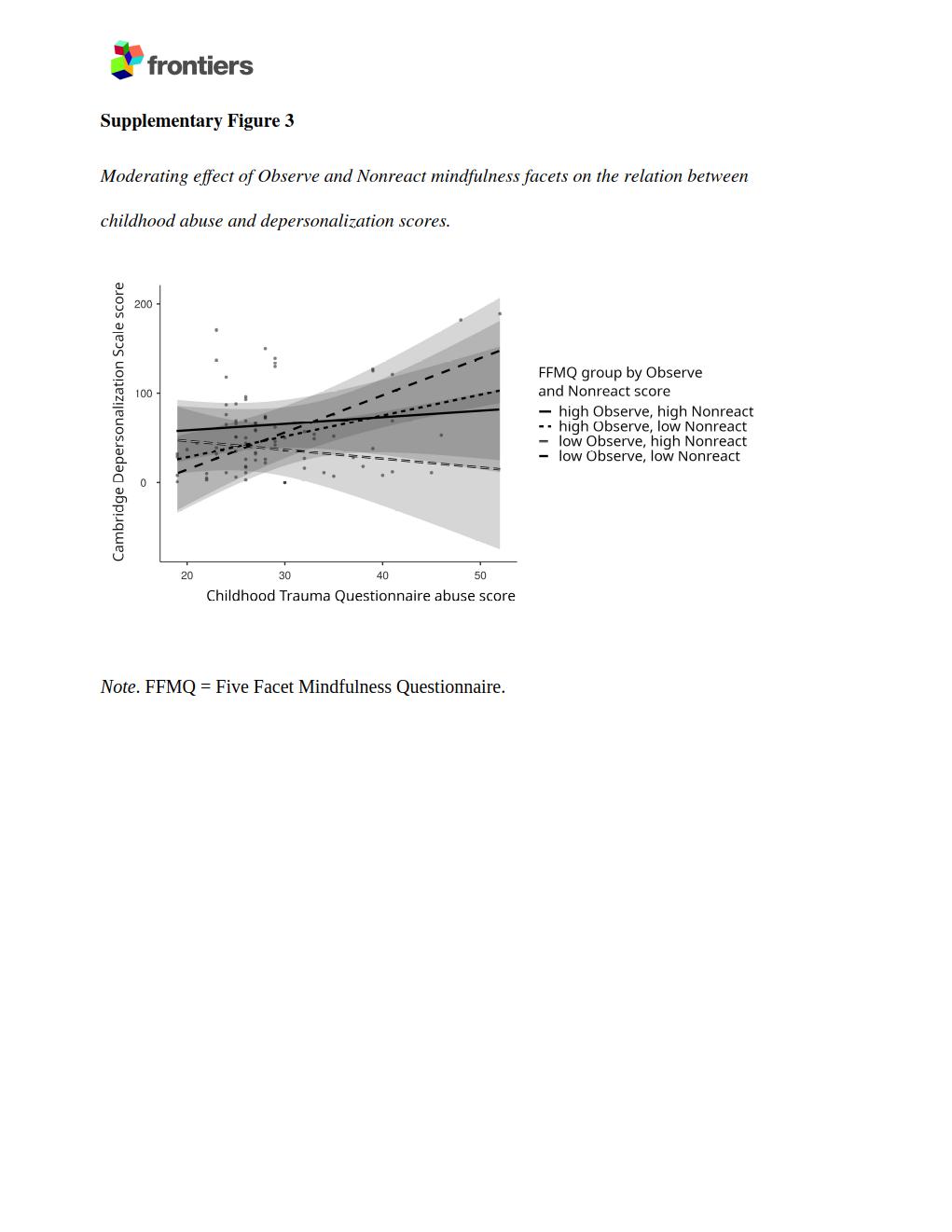

Supplement: Supplementary file 3 [file Image_3.JPEG]

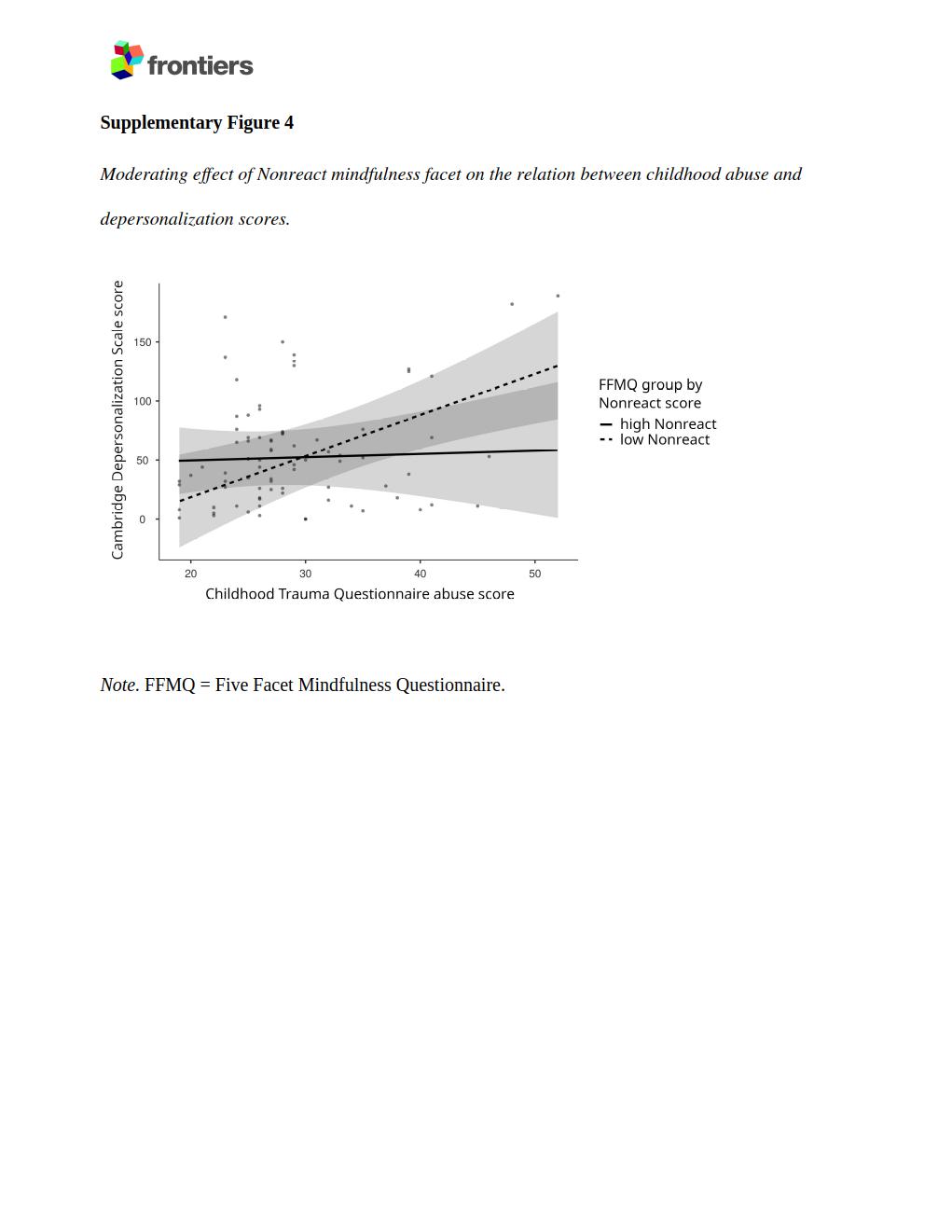

Supplement: Supplementary file 4 [file Image_4.JPEG]
