# Supplementary material for: Trait mindfulness buffers depersonalization symptoms among young adults exposed to childhood abuse
Source: Front Psychol. 2026 Jul 9;17:1838217. doi: 10.3389/fpsyg.2026.1838217 (PMC13391278; doi:10.3389/fpsyg.2026.1838217)
Supplement: Supplementary file 5 [file Table_1.DOCX]

| **Supplementary Table 1**  *Correlation matrix (Pearsons r) between primary variables and potential covariates.* | | | |
| --- | --- | --- | --- |
| ‍ | CTQ abuse score | FFMQ score | CDS score |
| CTQ abuse score | 1.00 | ‍ | ‍ |
| FFMQ score | -0.19 | 1.00 | ‍ |
| CDS score | **0.27*** | **0.30**** | 1.00 |
| Age (in years) | 0.05 | 0.14 | -0.19 |
| Sex | 0.02 | -0.11 | -0.03 |
| Race | 0.04^a^ | 0.04^a^ | 0.01^a^ |
| Hispanic or Latino | 0.08 | 0.08 | 0.04 |
| BMI | 0.16 | -0.05 | 0.03 |
| Depression, anxiety, or panic disorder diagnosis | 0.05 | -0.14 | 0.15 |
| Student status | -0.11 | -0.19 | 0.06 |
| *Note*. ^a^η^2^ value from ANOVA test. CTQ = Childhood Trauma Questionnaire; FFMQ = Five Facet Mindfulness Questionnaire; CDS = Cambridge Depersonalization Scale; BMI = Body Mass Index. ***p < .05, **p < .01.** | | | |
