# Supplementary material for: Trait mindfulness buffers depersonalization symptoms among young adults exposed to childhood abuse
Source: Front Psychol. 2026 Jul 9;17:1838217. doi: 10.3389/fpsyg.2026.1838217 (PMC13391278; doi:10.3389/fpsyg.2026.1838217)
Supplement: Supplementary file 6 [file Table_2.DOCX]

| **Supplementary Table 2**  *Multiple regression results for the prediction of depersonalization scores from childhood abuse score as moderated by trait mindfulness.* | | | | | | |
| --- | --- | --- | --- | --- | --- | --- |
| ‍‍‍‍‍‍‍ | *B* | standard error | β | *p* | *r^2^*_a(b,c)_ | *R^2^*_change_ |
| Block 1 | ‍ | ‍ | ‍ | ‍ | ‍ | **.04** |
| Age (in years) | -2.34 | 1.46 | -.17 | .114 | .027 | **‍** |
| Sex | -8.02 | 13.13 | -.06 | .543 | .004 | **‍** |
| Race | 1.65 | 2.11 | .09 | .437 | .006 | **‍** |
| Hispanic or Latino | -2.63 | 19.99 | -.01 | .896 | <.001 | **‍** |
| Block 2 | ‍ | ‍ | ‍ | ‍ | ‍ | **.17*** |
| CTQ abuse score | 1.30 | 0.71 | .20 | .071 | .035 | ‍ |
| FFMQ score | -22.96 | 9.32 | -.27 | .016* | .063 | ‍ |
| Block 3 | ‍ | ‍ | ‍ | ‍ | ‍ | **.24**** |
| CTQ abuse FFMQ | -3.09 | 1.23 | -.27 | .014* | .066 | ‍ |
| *Note.* CTQ = Childhood Trauma Questionnaire; FFMQ = Five Facet Mindfulness Questionnaire. ****p* < .05, ***p* < .01.** | | | | | | |
